# Validated Reverse-Phase High-Performance Liquid Chromatography for Quantification of Furosemide in Tablets and Nanoparticles

**DOI:** 10.1155/2013/207028

**Published:** 2013-09-16

**Authors:** Ibrahima Youm, Bi-Botti Celestin Youan

**Affiliations:** Laboratory of Future Nanomedecines and Theoretical Chronopharmaceutics, Division of Pharmaceutical Sciences, University of Missouri, Kansas City, MO 64110, USA

## Abstract

A simple, sensitive, and specific method for furosemide (FUR) analysis by reverse-phase-HPLC was developed using a Spherisorb C_18_ ODS 2 column. A chromatographic analysis was carried out using a mobile phase consisting of acetonitrile and 10 mM potassium phosphate buffer solution: 70 : 30 (v/v) at pH 3.85, at a flow rate of 1 mL*·*min^−1^. The UV-detection method was carried out at 233 nm at room temperature. Validation parameters including limit of detection (LOD), limit of quantitation (LOQ), linearity range, precision, accuracy, robustness, and specificity were investigated. Results indicated that the calibration curve was linear (*r* ^2^ = 0.9997) in the range of 5.2 to 25,000 ng*·*mL^−1^, with **ε** value equal to 3.74 × 10^4^ L*·*M^−1^ *·*cm^−1^. The LOD and LOQ were found to be 5.2 and 15.8 ng*·*mL^−1^, respectively. The developed method was found to be accurate (RSD less than 2%), precise, and specific with an intraday and interday RSD range of 1.233–1.509 and 1.615 to 1.963%. The stability of native FUR has also been performed in simulated perilymph and endolymph media (with respective potency in each medium of 99.8 ± 2.3% and 96.68 ± 0.7%, *n* = 3) after 6 hours. This method may be routinely used for the quantitative analysis of FUR from nanocarriers, USP tablets and release media related to hearing research

## 1. Introduction

**Figure 1:**
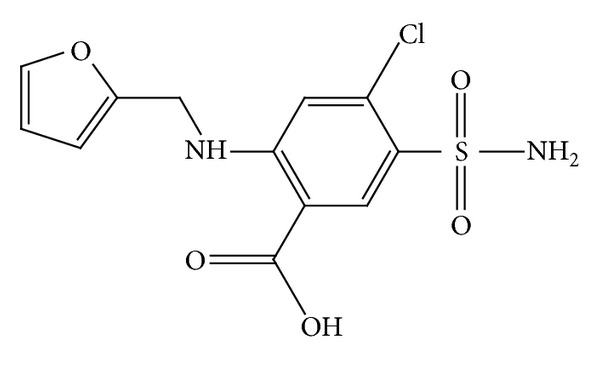
Chemical structure of furosemide.

Furosemide, 5-(aminosulfonyl)-4-chloro-2-[(2 furanylmethyl) amino] benzoic acid, (FUR) a loop diuretic has been used in the treatment of congestive heart failure and edema (Figure 1). FUR acts on thick ascending limb of the loop of Henle leading to a loss of sodium, potassium, and chloride that are dispatched in the urine [1]. This results in a decrease in sodium and chloride reabsorption, while increasing the excretion of potassium in the distal renal tubule. The diuretic effect of orally administered FUR appears within 30 minutes to 1 hour and is maximal in the first or second hour [2]. For the treatment of cardiac diseases, its daily dose is 20–80 mg for adults [3]. For pediatric use this dose is ranged from 1 mg·kg^−1^ up to a maximum of 40 mg daily neonates [3]. 

FUR has been reported to be the etiologic agent responsible for the permanent sensorineural hearing loss [4]. Recent work has demonstrated that FUR decreases the active cochlear mechanics in reducing the threshold shift following broadband continuous noise [5]. Thereby, it reduces the mobility of the basilar membrane of the cochlea and decreases the transduction that normally results from the bending of stereocilia on hair cells [6]. Therefore, FUR can be an appropriate drug model to target the inner ear. It can be also a potential approach for an understanding of the molecular mechanism of ototoxic drugs [7, 8]. The foregoing facts indicate that an extensive use of FUR results in frequent ototoxicity in humans. 

In mice, the total average of the inner ear volume is about 9.7 ± 0.2 *μ*L [9]. In guinea pig, the cochlear perilymph volume is reported to be 15.94 *μ*L [10]. Among the primate order, humans have significantly higher cochlear labyrinth volumes. The average volume of the inner ear fluid is reported to be 80.2 *μ*L [11, 12]. In addition to the small volume of the cochlear fluid, a small change of the cochlear fluid volume can impact drastically the high sensitivity of the hearing function. Therefore, the development and validation of an analytical method for FUR determination in biological fluid with a small sampling volume are urgently and critically needed for laboratory scale research.

Several methods including titrimetric, optical and electrochemical detections, and capillary electrophoresis have been used to quantify FUR [13]. Liquid-liquid extraction, followed by solvent evaporation, has been used for sample preparation in the chromatographic analysis of diuretics and probenecid in biological samples. Several reports addressed unresolved issues of precision, time consuming and loss of compounds of interest [14, 15]. Other researchers have proposed the use of micellar liquid chromatography for the determination of diuretics, such as FUR in pharmaceutical preparations with an elution time less than 18 min at a flow rate of 1 mL·min^−1^ [16]. The literature survey revealed that HPLC has been employed to detect FUR in blood, urine, or perilymph [17–19]. Recently, FUR has been simultaneously determined with spironolactone using HPLC [20]. So far, HPLC is generally the method of choice for diuretics quantitation, due to the required time and cost of the analysis [21]. 

In this paper, the development and validation of a reproducible RP-HPLC method intended to quantify FUR for routine laboratory use are described, according to the International Conference of Harmonisation (ICH) guidelines [22]. 

## 2. Materials and Methods

### 2.1. Chemicals and Reagents

Furosemide, potassium phosphate monobasic (KH_2_PO_4_), calcium chloride dehydrate, sodium chloride, potassium chloride, sodium bicarbonate, and potassium bicarbonate were purchased from Sigma-Aldrich (St. Louis, MO). Furosemide (USP tablets 20 mg) was provided by Mylan Inc (Canonsburg, PA). Dichloromethane was obtained from ACROS Organics (Morris Plains, NJ). Acetonitrile (HPLC grade) and formic acid were obtained from Fisher Scientific (Pittsburgh, PA). Poly-*ε*-caprolactone-polyethylene glycol (PCL-PEG, 2k-3k) diblock was supplied by advanced material polymers Inc (Montréal, Canada). Poly (D, L-lactic-co-glycolic acid) 50 : 50 (inherent viscosity, 0.4, 0.58, and 0.8 dL/g) were obtained from Birmingham Polymers (Pelham, AL). 

### 2.2. Equipment and Chromatographic Conditions

The liquid chromatographic system included a Waters-1525 Binary HPLC pump, a Waters 717 Plus autosampler, and a Waters 2487 dual *λ* absorbance detector (Waters Corporation, Milford, MA). The chromatographic analysis of FUR was performed under isocratic elution, using a Spherisorb ODS2 Column, C_18_, 80 Å, 5 *μ*m, 4.6 mm × 150 mm (Spherisorb, Queensferry, UK), a mobile phase composed of acetonitrile, and 10 mM potassium phosphate buffer (pH = 3.85) in a ratio 70 : 30 (v/v). Measurements were performed at 233 nm at a flow rate 1 mL·min^−1^ with an injection volume of 10 microliters. The analyte peak was confirmed by its characteristic retention time of 7.2 min. The mobile phase was degassed by sonication (Model 150 D, VWR, Minneapolis, MN) and filtered through a 0.22 *μ*m membrane filter (Whatman, UK). All the above chromatographic parameters were controlled by Waters Breeze software version 3.3 (Waters Corporation, MA).

### 2.3. Preparation of Stock and Working Standard Solutions

The primary standard solution of FUR was prepared with the mobile phase at a concentration of 25,000 ng·mL^−1^. The latter solution was further diluted with the mobile phase using a serial dilution method to obtain mixed working standard solutions from 5.2 to 25,000 ng·mL^−1^.

### 2.4. Method Validation

The validation method was carried out according to the ICH guidelines [22]. The present work entails evaluation of the following validation characteristics: limit of detection (LOD) and limit of quantitation (LOQ), specificity, linearity, precision, accuracy, recovery, and robustness.

#### 2.4.1. Specificity

The objective of this study was to measure the FUR amount in the occurrence of UV interference in the presence of excipients that may be expected to be present in the nanocarrier (NC) matrix extract. The procedure used to demonstrate the specificity depended on the intended objective of the analytical method. For this purpose, the analyte discrimination was carried out in the presence of the excipients (poly-*ε*-caprolactone-polyethylene glycol (PCL-PEG) and poly (D, L-lactic-co-glycolic acid) (PLGA)) used for the NC preparation. A small amount of FUR was spiked with appropriate amount of excipients. Three samples of placebo were prepared by dissolving 1, 2, and 3 mg of powdered blank NCs in 1.5 mL of a mixture of acetonitrile/dichloromethane (14 : 1 v/v) for 20 min at room temperature. Three samples were centrifugated at 15,000 rpm for 15 min (Micro 18R, VWR, Minneapolis, MN). Finally, a volume of 40 *μ*L of the supernatant was diluted (7 times). Samples were analyzed by HPLC taking into account the lowest limit of quantification.

#### 2.4.2. Detection Limit and Quantitation Limit

The objective of the determination of the LOD was to establish the minimum level from which an amount of FUR can be reliably detected. The determination of the LOQ aimed to detect the minimum injected amount of drugs that produces quantitative measurements with acceptable precision. The LOD and LOQ were determined using the standard deviation of the response and the slope of the calibration curve. The samples were diluted in the mobile phase by serial dilution and analyzed in triplicate. The calibration curve was constructed using drug concentrations (5.2, 62.5, 250, 700, 3,500, 14,000, and 25,000 ng·mL^−1^). The LOD and LOQ were obtained from (1) and (2) considering Beer-Lambert's law:

(1) $$\begin{array}{c} \text{LOD}=\frac{3.3\sigma }{S}, \end{array}$$

(2) $$\begin{array}{c} \text{LOQ}=\frac{10\sigma }{S}, \end{array}$$

where *σ* is the standard deviation of the chromatogram peak area containing the analyte in the range of the LOD or the LOQ and *S* is the slope of the calibration curve [22].

#### 2.4.3. Accuracy and Precision

The purpose of this assay was to verify whether the method will provide the same results in three different concentrations and on three different days. 

 The precision was determined by repeatability (intraday) and intermediate precision (interday) with standard quality control samples. The accuracy was evaluated by replicating (*n* = 5) the analysis of the samples from three different concentrations of FUR: 5, 7.5, and 10 *μ*g·mL^−1^ (50, 75, and 100%). The deviation of the mean from the true value serves as the measure of accuracy and should be less than 15% [22].

The percent of FUR recovered from a known amount was carried out using data from 5 corresponding responses per day on three consecutive days over the three concentrations listed above. The amount of FUR was estimated by measuring the peak areas and by fitting these values to the straight-line equation of the calibration curve. At each concentration level, the precision should not exceed 15% [22]. Both accuracy and precision were reported as %RSD for statistical significance.

#### 2.4.4. Linearity of the Calibration Curve

This study aimed to monitor the relationship between the response (area under curve) and the concentration of FUR. Linearity was evaluated by using seven standard concentrations of FUR ranging between 5.2, 62.5, 250, 700, 3,500, 14,000, and 25,000 ng·mL^−1^ and analyzed in triplicate. The calibration curve was obtained by plotting the peak area to the spiked theoretical concentrations of FUR from the standard solutions and the regression equations were calculated. Seven samples were quantified using the concentration peak area relationships. The linearity of the calibration curve was evaluated by linear regression analysis. For acceptance, a correlation coefficient (*r* ^2^) of 0.99 or better was required [22].

#### 2.4.5. Recovery

The extraction efficiency of FUR was quantified at three different concentrations, 5,000, 7,000 and 9,000 ng·mL^−1^, as low-, medium-, and high-quality control samples (50, 70, and 90% w/v, resp., *n* = 3). To prepare the sample solution (assay of pharmaceutical preparation), ten tablets (20 mg FUR, USP) were weighed individually to obtain afterwards the average weight. After grinding these tablets with a mortar and a pestle, an equivalent amount of 20 mg of FUR was weighed. 

 On the other hand, an equivalent amount of FUR-loaded NCs (20 mg) was used in parallel. Both powders were dissolved in a 20 mL volumetric flask, containing 10 mL of ethanol, and sonicated for 10 min to accelerate the dissolution process. The samples were transferred in 2 mL microcentrifuge tubes (Sigma Aldrich, Saint-Louis, MO) and centrifuged at 15,000 rpm for 15 min. Then, 75 *μ*L of each solution was aliquoted in 2 mL microcentrifuge tubes and diluted in 1.425 mL of mobile phase to obtain a total volume of 1.5 mL. The extraction recovery of FUR was determined by comparing the obtained chromatogram peak areas with unextracted standards representing 100% recovery (*n* = 3). 

#### 2.4.6. Robustness

The purpose of this test was to demonstrate that the method performance would not be significantly impacted by slight variations of the method parameters. By means of JMP software, version 8 (SAS Institute, Cary, NC), a fractional factorial design (2^*k*−1^) was constructed with the aim of calculating the effects of the main chromatographic parameters including acetonitrile/phosphate buffer ratio (*X*_1_), wavelength (*X*_2_), pH (*X*_3_), and flow rate (*X*_4_) on the chromatogram peak area. A two-level fractional factorial design (2^4−1^) was used to evaluate the influence of selected parameters as shown in Table 1. Each experiment was performed in replicate (*n* = 5) and the average was used for computational analysis.

### 2.5. Determination of the Plate Number

In order to determine the column efficiency, the plate number (*N*) was calculated using (3)

(3) $$\begin{array}{c} N=\frac{3000\times L}{d_{P}}, \end{array}$$

where *L* is the column length in centimeter and *d*_*P*_ is the packing particle diameter in micrometer.

### 2.6. Application Method

This method was applied for the determination of FUR-loaded in polymeric biodegradable NCs and USP tablets. In our previous works, FUR-loaded nanocarriers were successfully synthesized and physicochemically characterized [24]. In this present work, the same method is used to estimate the percent of FUR encapsulation efficiency in nanocarriers. 

#### 2.6.1. Determination of the Molar Absorptivity of Furosemide by UV-Spectrometry Method

The *ε* value of FUR was calculated by preparing a calibration curve in the concentration range of 1,000–25,000 ng·mL^−1^, using a UV-Vis spectrophotometer at 233 nm. For each concentration, the absorbance was calculated and a plot was constructed by putting the absorbance on the *y-*axis and the concentration on the *x*-axis. The linear regression data analysis was performed through the experimental points. Setting the intercept of the calibration curve to zero allowed us to obtain the *ε* value from the slope of this curve by using the classic Beer-Lambert law:

(4) $$\begin{array}{c} A=\varepsilon \cdot l\cdot c, \end{array}$$

where the absorbance (*A* or Abs) of a substance depends on the path length (*l*), that is, the length of solution through which the light passes. For our cells, *l* = 1 cm and the concentration (*c*) was expressed in moles·L^−1^ of the substance.

#### 2.6.2. Determination of the Molar Absorptivity of Furosemide by HPLC Method

The developed HPLC method was applied for the estimation of the *ε* value of FUR. For the calculation of the *ε* by HPLC, (4) was rearranged to get the following [25]:

(5) $$\begin{array}{c} A_{r}=\frac{0.06\times l\times \varepsilon \;\text{ng}}{M\times f}, \end{array}$$

where *A*_*r*_ is the area of the drug chromatogram (mAU·s) recorded by the UV detector at 233 nm, *l* is the path length (cm), *M* is the molecular weight of the drug (Dalton), *f* is the mobile phase flow rate (mL·min^−1^), and ng (in nanogram) is the injected drug amount for each injection volume of ten microliters. The elaborated (5) could be used as an alternative tool to better estimate the *ε* value of any molecule using HPLC data. In this equation, all other parameters, except *A*_*r*_ and *ε*, were constant for a given drug. Thus, they can be referred to as a constant “*k*” leading to

(6) $$\begin{array}{c} A_{r}=\varepsilon \cdot k. \end{array}$$

In this method, *ε* was estimated using the slope of the regression curve generated from the injected amounts and other constants from (5), leading to *k* (nmol·Min·mL^−1^·L^−1^) and the corresponding areas (*A*_*r*_).

#### 2.6.3. Short-Term Stability

This experiment was performed by simulating the cochlear fluid's composition as shown in Table 2 [23]. An accurately weighed quantity of native FUR (50 *μ*g) was transferred into 50 mL of perilymph or endolymph simulated fluid (in accordance with the sink conditions) and incubated in triplicate at 37 ± 0.2°C in constant stirring for 6 hours. Within this time, samples were taken at each established sampling time (every hour). The withdrawn aliquots (40 *μ*L) were diluted with pure water (7-fold) in order to obtain a final concentration in the range of the calibration curve. Then, the resulting samples were analyzed using the above HPLC method.

### 2.7. Statistical Analysis

A *t*-student test was applied to compare the means of two independent samples. A *P* value (*P*) below 0.05 was considered statistically significant. A polynomial equation of the chromatogram area response values was derived from the results of the eight runs in the fractional factorial design.

## 3. Results and Discussion

### 3.1. Method Development

The aim of this work was to develop a suitable and reproducible new analytical method and routinely determine the amount of FUR. The selection of the chromatographic conditions was based on minimizing the tailing factor, improving the peak symmetry, and reducing the total analysis time. Therefore, a wavelength scanning was first performed in the range of 200–400 nm using the UV-Vis spectrophotometer. The maximum absorbance of FUR with adequate sensitivity was found to be 233 nm.

To obtain the best ratio of acetonitrile/phosphate buffer (A/P), experiments were carried out with an A/P ratio ranging from 10 to 90% v/v. The best A/P ratio was found to be 30 : 70 (v/v). To optimize the mobile phase composition, acetonitrile and 0.01 M potassium phosphate buffer were prepared at different pHs (3.0, 3.5, 4.0, 5.0, and 6.0) using formic acid as pH modifier. At pH 3.0, the peak resolution was found to be low. From pH 4.0 to 6.0, the tailing factor of the FUR peak was poor (1.6 to 1.88). 

The best separation of the analyte with high efficiency, peak symmetry, and reproducibility was achieved with a mobile phase composition of A/P; 30 : 70, v/v at pH 3.85. FUR is a rather poorly water-soluble drug (acidic pKa = 3.22) and contains carboxylic acid group, which is ionized to a great extent (pH > pKa) [26, 27]. The relationship between pKa and pH and their importance in buffer preparation is known as the classical Henderson-Hasselbalch equation [28, 29].

 As indicated, the retention time (7.2 min) was reproducible (RSD value equal to 0.31%) and consistent with published data [30]. In addition, the symmetry factor 1.29 was lower than the upper limit (1.5) according to ICH guidelines and consistent with previous results [31]. From the literature, there is no available data about the plate number of column in FUR quantitation by HPLC. The calculated plate number (*N* value using (3)) was found to be 9000 and fulfill the theoretical requirement of the *N* value (*N* ≥ 1,000, [32]). 

### 3.2. Method Validation

The present method was validated according to ICH guidelines [33]. The method validation was performed in terms of specificity, accuracy, precision, LOD, LOQ, recovery, linearity, and robustness.

#### 3.2.1. Specificity

**Figure 2:**
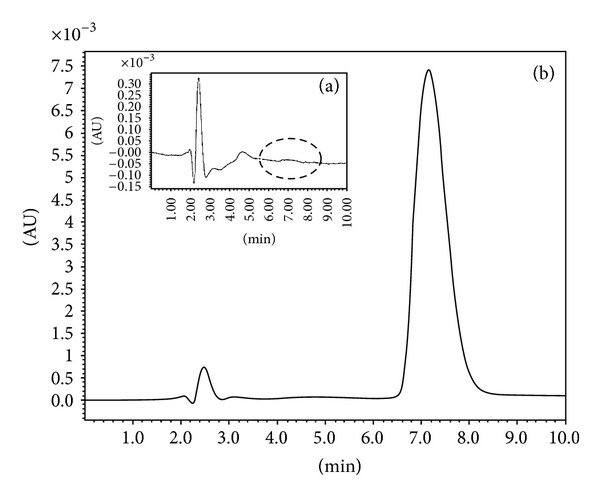
Chromatogram of (a) extracts from blank nanocarriers and (b) mixture of 25 *μ*g/mL of FUR (w/vol) with blank nanocarriers.

The ability of the method to discriminate the analyte in the presence of the excipients used for the NC preparation was carried out. These excipients included poly-*ε*-caprolactone-polyethylene glycol (PCL-PEG) and poly (D, L-lactic-co-glycolic acid, PLGA). Blank NCs ranged from 106 to 400 nm in diameter were used to check the specificity of the optimized method. No interfering peak of endogenous compounds around the retention time of FUR was found at 233 nm (Figure 2(a)). The result showed also an absence of interference of the commercial grade FUR tablets. A well-defined peak at the retention time of FUR was shown, when a known amount of FUR was spiked into the previous colloidal suspension of blank NCs (Figure 2(b)). Therefore, the optimized method is suitable for the detection and quantification of FUR [22].

#### 3.2.2. Detection and Quantitation Limits

The method sensitivity was examined through the determination of the LOD and LOQ using the signal-to-noise approach. LOD was found to be at 5.2 ng·mL^−1^ corresponding to a concentration of 0.019% of the working solution (25,000 ng·mL^−1^, (1)). The obtained LOD was lower than those found previously in the literature [17, 34, 35]. The quantitation limit was found to be 15.8 ng·mL^−1^ (2) corresponding to 0.056% which is lower than 1% FUR peak concentration (25,000 ng·mL^−1^). Both LOD and LOQ fulfill the accepted criteria (<10%). 

#### 3.2.3. Accuracy and Precision

The accuracy of the analytical method was assessed by comparing a known concentration of FUR to the experimental value. The relative standard deviation (RSD%) of the intraday precision varied from 1.233 to 1.509% (Table 3), which complies with the acceptance criteria proposed by the ICH guideline (RSD < 2.0%). However, the precision was evaluated at the above concentration levels for 3 days with RSD% between 1.615 and 1.963%. At this concentration range, the upper limit was in the limit of acceptance criteria of ICH guideline [22].

#### 3.2.4. Linearity and Range

The linearity of the relationship between the measured and theoretical drug concentrations was investigated. The measured concentration of FUR was obtained from the linear regression analysis of the calibration curve. A typical calibration curve of FUR peak area within a concentration range of 5.2 to 25,000 ng·mL^−1^ was obtained with the following linear regression line: *Y* = 54,061*X*− 1,769.2 (*r* ^2^ = 0.9997). The latter equation confirms also that *y*-intercept at zero concentration of FUR was not negligible.

#### 3.2.5. Recovery

FUR from nanocarriers and tablets was quantified at concentrations of 5, 7, and 9 *μ*g/mL (50, 70, and 90% w/w, resp.). These data fulfilled the acceptance ICH criteria (Table 4, RSD < 2%) and were consistent with previous study [36].

#### 3.2.6. Robustness

The robustness of a method is an indication of its reliability during the analytical procedure. A fractional factorial design (2^*k*−1^) was constructed with the aim of calculating the effects of a deliberate change of the main chromatographic parameters (*X*_*i*_) on the chromatogram peak area (*Y*). *X*_1_ represented the percentage of acetonitrile (v/v) in the mobile phase, *X*_2_ was the UV wavelength, *X*_3_ was the pH of the mobile phase, and *X*_4_ was taken as the flow rate of the mobile phase. As expected, the chromatogram peak area decreased when the content of acetonitrile was increased from 28 to 32% (v/v).

**Figure 3:**
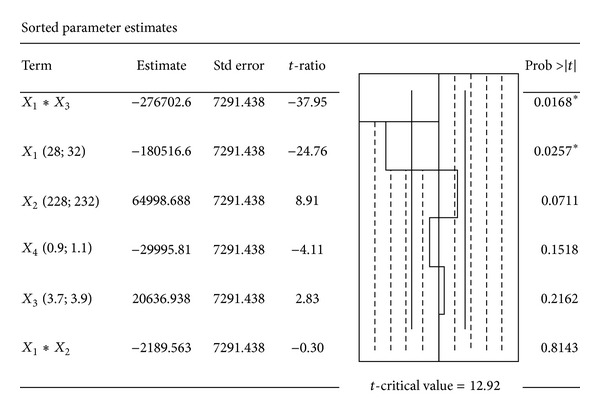
Pareto chart showing the effects of HPLC parameters on the chromatographic peak area.

 The Pareto chart of the effects of chromatographic parameters on the peak area was shown in Figure 3. A negative sign of the *t*-ratio indicated a negative effect of the chromatographic parameter on the *Y* value, while a positive sign of the *t*-ratio indicated a positive effect of the chromatographic parameter on the *Y* value. It was clearly shown that *X*_1_ and its interaction with *X*_3_ had a statistically significant negative impact on the *Y* value (*P* = 0.0257 and 0.0168, resp.). This seemed to indicate that an increase of the volume of acetonitrile in the mobile phase may lead to underestimate the drug quantification. This result can be attributed to a weakness of the buffer strength of the mobile phase when the acetonitrile volume is increased. Indeed, at pHs above the pKa of FUR, the acidic group of the analyte will be negatively charged, thus increasing its polarity. Therefore, an adequate retention would be achieved in adding a small volume aqueous mobile phase, knowing that the retention time of such hydrophobic analyte may also be extended [37, 38]. 

However, *X*_2_, *X*_3_, and *X*_4_ had no significant effects on the *Y* value (*P* = 0.0711, 0.2162, and 0.1518, resp.). This appeared to indicate that the method was enough robust at these working conditions. For a better use of the validated method, the percent of acetonitrile and its interaction with the mobile phase pH should be carefully controlled.

### 3.3. Application Method

#### 3.3.1. Calculation of the Molar Absorptivity (*ε*)

The calibration curves constructed in the concentration ranged defined in Table 6 for the determination of the *ε* value using a UV-Vis spectrophotometer and HPLC were found to be linear with correlation coefficient (*r* ^2^ = 0.999 and 0.996, resp., Table 5). Prior to this application method, a calibration curve was developed for the analysis of FUR. 

By applying a UV spectrophotometric procedure, a regression analysis of Beer Lambert's law showed a good correlation in the concentration ranged defined in Table 6 with a molar absorptivity of 3.66 × 10^4^ 141.27 ± L·M^−1^·cm^−1^ (*n* = 3). 

By applying the HPLC method, the results indicated that the calculated molar absorptivity was 3.74 × 10^4^  ± 398.74 L·M^-1 ^cm^−1^ (*n* = 3), Table 6. Using the Student *t*-test, the difference between these two values (both from UV spectrophotometry and HPLC) was not statistically significant (*P* = 0.9975) and not consistent with previous report [39].

#### 3.3.2. Stability Test

**Figure 4:**
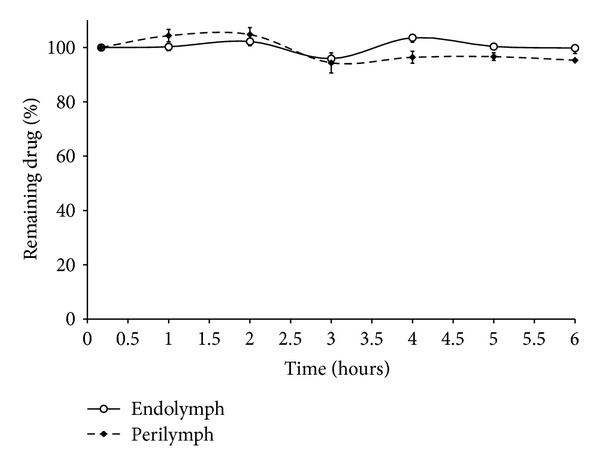
Stability of FUR in simulated cochlear fluid (*n* = 3).

Preliminary study in the author's laboratory indicated that FUR's solubility increased in simulated endolymph and perilymph media: 500,000 ng·mL^−1^ and 41,000 ng·mL^−1^, respectively versus 6,000 ng·mL^−1^ in water (unpublished data). Indeed, the increased pH value of the medium improved the solubility of FUR. Also the degree of ionization may be responsible for the increase in solubility in the cochlea simulated media. The obtained results (from simulated perilymph and endolymph) showed that FUR (50 *μ*g) was stable over the course of the experiment (6 hours). The obtained data indicate a minimum decrease of the drug concentration below the minimum percentage (95%, Figure 4). Bearing in mind the small volume of the cochlear compartment and its sensitivity to small change in volume, the developed HPLC method could be useful by filling this compartment with 40 *μ*L simulated perilymph (0.5 *μ*L·min^−1^) after collecting 40 *μ*L of perilymph sample.

## 4. Conclusion

The present HPLC method was exhibited acceptable to have sensitivity, linearity, precision, robustness, low limit of detection, and quantitation according to the ICH guideline. Results from the developed method provided satisfactory outcomes with low level of detection, good accuracy, small sampling volume, and low degree of interference of inactive ingredients from NCs and tablets. The method application allowed the determination of the FUR EE% from the NCs with accurate results. A good estimation of the molar absorptivity and FUR stability in simulated cochlea fluid can be obtained using the validated method. Due to the widespread use of FUR resulting in ototoxicity concerns, an increasing interest in FUR quantitation in inner ear fluids could be expected in the near future. 

## Figures and Tables

**Table 1:** Experimental design matrix for the robustness study and response values (*n* = 5).

Pattern	Acetonitrile percent in water (%) v/v	Wavelength (nm)	pH	Flow rate (mL·min^−1^)
	*X*_1_	*X*_2_	*X*_3_	*X*_4_
+−−+	32	228	3.7	1.1
++−−	32	232	3.7	0.9
−++−	28	232	3.9	0.9
++++	32	232	3.9	1.1
−+−+	28	232	3.7	1.1
−−−−	28	228	3.7	0.9
−−++	28	228	3.9	1.1
+−+−	32	228	3.9	0.9
Low level	28	228	3.7	0.9
High level	32	232	3.9	1.1

**Table 2:** Chemical composition of the synthetic cochlear fluids without protein (data summary adapted from Wangemann and Schacht) [23].

Ion	Perilymph synthetic media (*Scala vestibuli*)	Endolymph synthetic media
Na^+^	141 mM	1.3 mM
K^+^	6 mM	157 mM
Cl^−^	121 mM	132 mM
Ca^2+^	0.6 mM	0.023 mM
CO^3−^	18 mM	31 mM
pH	7.3	7.4

**Table 3:** Interday and intraday assay reproducibility in aqueous solutions of FUR (*n* = 5).

Spiked FUR concentration (ng/mL)	Intraday			Interday		
	Mean calculated concentration (*μ*g/mL)	SD*	RSD%	Mean calculated concentration (*μ*g/mL)	SD	RSD%
5,000	5.300	0.080	1.509	5.325	0.091	1.709
7,500	7.464	0.092	1.233	7.429	0.120	1.615
10,000	10.820	0.156	1.442	10.852	0.213	1.963

*Standard deviation.

**Table 4:** Determination of the percent encapsulation efficiency (EE%, ± SD) in USP tablet and FUR-loaded nanocarriers (*n* = 3).

Percent of theoretical drug amount (%)	FUR-loaded nanocarriers		Furosemide tablet USP	
	(%) Recovery	RSD%	(%) Recovery	RSD%
90	90.84 ± 1.77	1.95	88.26 ± 1.75	1.98
70	69.32 ± 0.89	1.28	68.72 ± 1.18	1.71
50	49.56 ± 0.23	0.47	50.23 ± 0.74	1.47

**Table 5:** Data from linear standard curves, obtained separately by plotting *Y* versus *X*.

	Number of points	Range	Correlation coefficient (*r* ^2^)	Equation
a	5	0–2 mol·L^−1^	0.9989	Abs = 36,501·*C *
b	7	0–0.05 nmol·min·mL^−1^·L^−1^	0.9956	Area = 37,357·*K *

^a^Absorbance (*Y*, AU) versus FUR concentration (*X*, mol·L^−1^).

^b^Area of chromatogram (*Y*, mAU·s) versus *K* (*X*, nmol·min·mL^−1^·L^−1^).

**Table 6:** Determination of the molar absorptivity by UV spectrophotometry and HPLC.

Method	Calibration range (ng/mL)	Equation	*r* ^2^	Slope (*ε*)	Mean	Relative standard deviation (%)
UV	1,000–25,000	Abs = 36,745*k*	0.996	36,745		
		Abs = 36,506*k*	0.992	36,506		
		Abs = 36,495*k*	0.998	36,495	36,582	0.386

HPLC	1,000–25,000	Area = 37,023*c*	0.998	37,023		
		Area = 37,819*c*	0.995	37,819		
		Area = 37,379*c*	0.993	37,379	37,407	1.065
